# Risk of retinal disease and visual impairment in individuals with psychiatric disorders

**DOI:** 10.1038/s41433-025-03851-w

**Published:** 2025-05-20

**Authors:** Jeffrey Chu, Jacqueline K. Shaia, Hejin Jeong, Rishi P. Singh, Katherine E. Talcott

**Affiliations:** 1 Case Western Reserve University School of Medicine, Cleveland, OH USA; 2 Center for Ophthalmic Bioinformatics, Cole Eye Institute, Cleveland Clinic, Cleveland, OH USA; 3 Cleveland Clinic Martin Hospitals, Cleveland Clinic Florida, Stuart, FL USA; 4 Cleveland Clinic Cole Eye Institute, Cleveland, OH USA; 5 Cleveland Clinic Lerner College of Medicine of Case Western Reserve University, Cleveland, OH USA

**Keywords:** Epidemiology, Eye diseases

## Abstract

**Background/Objectives:**

Individuals with a psychiatric disease have been reported to have structural variations in the retina, but how this affects retinal disease risk and vision loss is poorly understood. This study evaluated the risk of retinal disease and visual impairment in individuals with psychiatric disorders.

**Subjects/Methods:**

An exploratory retrospective cohort study was conducted through a federated health research network that aggregates de-identified EHR data of over 95 million individuals across 50 healthcare organizations. Individuals ages 50–89 were identified for schizophrenia, bipolar disorder (BD), major depressive disorder (MDD), retinal disease, and visual impairment defined by vision loss or blindness using ICD-10 codes. Individuals were propensity score matched (PSM) on age, sex, race, ethnicity, hypertension, diabetes, and dyslipidaemias. Risk ratio calculation and statistical analyses were conducted through the network’s analytics tool utilizing 95% confidence intervals.

**Results:**

After PSM, the schizophrenia cohort had 160,414 matched individuals (average age 65), 391,440 in the BD cohort (64), and 1,962,380 in the MDD cohort (67). A recorded diagnosis of schizophrenia was associated with a decreased likelihood of having a retinal disease diagnosis, while recorded diagnoses of BD and MDD were associated with an increased likelihood. Across all psychiatric disorders, individuals with a retinal disease diagnosis had an increased risk of visual impairment compared to individuals with a retinal disease alone.

**Conclusion:**

Recorded diagnoses of BD and MDD were associated with an increased likelihood of having a retinal disease diagnosis. Across all psychiatric disorders, individuals with a concurrent retinal disease were more likely to have a visual impairment diagnosis.

## Introduction

Psychiatric disorders, including schizophrenia, bipolar disorder (BD), and major depressive (MDD), are chronic conditions that are detrimental to individual health and well-being. These disorders not only impose a substantial negative impact on the worldwide burden of disability and life satisfaction but have also been associated with increased risks of suicide, premature mortality, and modifiable coronary risk factors, such as obesity, smoking, diabetes mellitus, and hypertension [1–5]. The World Health Organization (WHO) has reported that the prevalence of psychiatric disorders worldwide has increased by more than 13% since 2007 and is only expected to rise [6].

The relationship between cerebral and ocular disease has been demonstrated to be one of significant clinical utility. Because the brain and the retina both develop from the neuroectoderm, they share many similar morphological and physiological properties. Therefore, changes within the retina may reflect pathological changes in the brain that are associated with cerebral disease. This allows the eyes to serve as a “window” to evaluate disorders originating from the brain [7]. Previous investigations have already found that retinal vascular change or degeneration was associated with Alzheimer’s and Parkinson disease, suggesting that neurological diseases impact ocular health and therefore these individuals should be screened and monitored for ocular manifestations [8, 9].

In addition to neurodegenerative disorders, psychiatric disorders have also been linked to changes within the eye, specifically the retina. Utilization of optical coherence tomography (OCT) in individuals with schizophrenia found notable retinal thinning and reduced macular thickness and volume [10–13]. In addition, OCT findings in individuals with BD have found significant global thinning of the peripapillary retinal nerve fiber layer (pRNFL) and lower ganglion cell layer (GCL) and inner plexiform layer (IPL) volume [14–16]. When evaluating individuals with MDD using OCT, Kalenderoglu et al. found that these individuals had significantly reduced GCL, IPL, and global and temporal superior RNFL thickness [17]. These findings cumulatively shed light on how psychiatric disorders affect the structural component of the retina. However, how these structural abnormalities might lead to functional abnormalities and ultimately, retinal disease and subsequent visual impairment has not been thoroughly investigated.

A nationwide cohort study in Taiwan that explored the risk of retinal disease in individuals with BD found that this population was associated with a higher risk of retinal detachments, primary retinopathy, hypertensive retinopathy, and retinal vascular complications [18]. This study was conducted via records from a national Taiwanese database, highlighting the need for further investigation to assess if these findings are generalizable to the United States. The literature on the relationship between psychiatric and retinal disease is sparse, with this study being the only one to date that has explored the risk of retinal disease in individuals with a psychiatric disorder. This study did have limitations by only focusing on individuals with BD, being a relatively smaller study cohort, and evaluating a limited number of retinal diseases. As it is difficult to obtain large sample sizes of individuals with these more uncommon disorders at a singular institution, aggregated electronic health record research networks provides us the unique opportunity for a large group exploratory analysis.

This purpose of this exploratory analysis was to examine the risk of retinal disease in individuals with psychiatric disorders, specifically schizophrenia, BD, and MDD within a large aggregate electronic health records network. Retinal disease was defined as retinal conditions with an associated ICD-10 code (Supplementary Table 1) such as H35.32 for neovascular age-related macular edema (AMD). Additionally, this study explored how the presence of a psychiatric disorder affects the risk of visual impairment in individuals with a retinal disease.

## Materials/subjects and methods

This population-based retrospective cohort study was conducted through the TriNetX Analytics Network, a federated health research network that aggregates the de-identified EHR data of over 95 million individuals across 50 healthcare organizations (HCOs). TriNetX, LLC is compliant with the Health Insurance Portability and Accountability Act (HIPAA), the US federal law which protects the privacy and security of healthcare data, and any additional data privacy regulations applicable to the contributing HCO. The process by which the data is de-identified is attested to through a formal determination by a qualified expert as defined in Section §164.514(b)(1) of the HIPAA Privacy Rule. Because this study used only de-identified patient records and did not involve the collection, use, or transmittal of individually identifiable data, this study was exempt from Western Institutional Review Board approval. Data was collected over 2 weeks in May 2023.

This study compared individuals with a recorded diagnosis of schizophrenia, bipolar disorder (BD), or major depressive disorder (MDD) (case) and those without such diagnoses (control) to assess whether they have different rates of receiving a diagnosis of retinal disease and visual impairment. The retinal diseases investigated were chronic or age-related conditions, such as diabetic retinopathy and age-related macular degeneration (AMD) and their associated complications (Supplementary Table 1). To target the population at the highest risk of developing these conditions and to allow sufficient time for these conditions to manifest, this study selected individuals aged 50 and older as the primary focus group [19, 20]. Individuals were evaluated up to age 89 because this database classifies individuals 90 and above as a protected population. The database was queried for any eligible individuals prior to a diagnosis index date of May 1^st^, 2023

All diagnoses data regarding psychiatric disorders, retinal disease, and visual impairment outcomes were extracted using International Statistical Classification of Diseases and Related Health Problems, Tenth Revision (ICD-10) codes. The ICD-10 codes used to determine the diagnoses of schizophrenia, BD, and MDD were F20, F31, and F32-33 respectively. The primary outcome measure of having a recorded diagnosis of a retinal disease was defined by the ICD-10 codes, E10.31-E10.35, E11.31-E11.35, H31.0, and H33-H36. The secondary outcome measure of having a recorded diagnosis of visual impairment was determined by the ICD-10 code, H54. Additionally, specific retinal conditions were investigated including diabetic retinopathy (DR), chorioretinal scars, retinal detachment, retinal vascular occlusions, neovascular AMD, non-neovascular AMD, macular cyst or hole, cystoid macular degeneration, degenerative drusen of macula, puckering of macula, peripheral retinal degeneration, retinal hemorrhage, separation of retinal layers, retinal edema. Retinal disease and visual impairment diagnoses data were collected at person level, with no reference to eye laterality. All ICD-10 codes for these conditions are listed in Supplementary Table 1.

In order to determine how a recorded diagnosis of schizophrenia, BD, or MDD may be associated with the risk of having a recorded diagnosis of a retinal disease, we queried the dataset of eligible individuals to create a case cohort and a control cohort—the cases had the diagnosis of a particular psychiatric disorder of interest while the controls did not. In addition, individuals were stratified by age to best explore if age is associated with earlier onset of retinal disease within the study population. Age stratification by decade was performed to create sub-cohorts that were later compared to determine the risk ratio (RR) of having a recorded diagnosis of a retinal disease in addition to specific retinal diseases. To determine how the colocalization of a psychiatric disorder with a retinal disease impacts a recorded diagnosis of visual impairment, we again queried the dataset of eligible individuals to create a case and control cohort – the case cohort had a recorded diagnosis of both a psychiatric disorder and retinal disease while the control cohort only had a history of a retinal disease but without a psychiatric disorder. For both sets of analyses, we provide an unadjusted RR in addition to an adjusted RR after propensity score matching (PSM).

One-to-one greedy matching algorithm with a calliper of 0.25 pooled standard deviations was utilized to perform PSM and incorporates multiple imputations for any missing pieces of data. Cohorts were matched on age, sex, race, ethnicity, hypertensive diseases (ICD-10 code: I10-I16), diabetes mellitus (E08-E13), and disorders of lipoprotein metabolism or dyslipidaemias (E78). The RR of having a recorded diagnosis of a retinal disease and visual impairment and their 95% confidence intervals (CI) were calculated and significant tests were 2-sided and paired. A significance threshold of 0.05 or less was used. All statistics analyses were conducted within the TriNetX analytics tool and forest plots were created in R Studio.

## Results

**Fig. 1:**
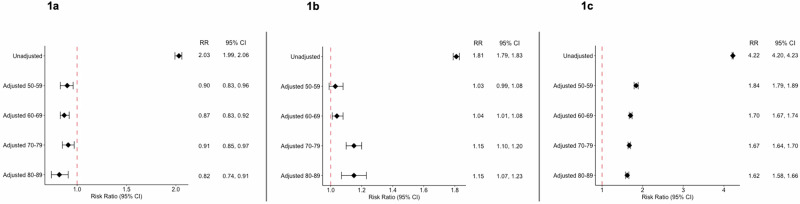
Relative risk of having a retinal disease diagnosis in individuals with a psychiatric disorder compared to individuals without a psychiatric disorder after age stratification and propensity score matching. **a** Schizophrenia; (**b**) BD; (**c**) MDD. BD bipolar disorder, MDD major depressive disorder, RR risk ratio, CI confidence interval.

At the time of this study, this network had a total population of 38,824,237 for individuals age 50–89. Average age was 65 for individuals with a recorded diagnosis of schizophrenia, 63 in individuals with a recorded diagnosis of BD, and 67 in individuals with a recorded diagnosis of MDD. Additional demographics information can be found in Supplementary Table 2. After propensity score matching in individuals with a recorded diagnosis of schizophrenia, there were 55,163 individuals in the age 50–59 cohort; 62,479 individuals in the age 60–69 cohort; 31,573 individuals in the age 70–79 cohort; and 11,199 individuals in the age 80–89 cohort. The unadjusted RR of having a recorded diagnosis of retinal disease in individuals with a recorded diagnosis of schizophrenia was 2.03 compared to individuals without a recorded diagnosis of schizophrenia (95% CI 1.99, 2.06). After age stratification and PSM, a recorded diagnosis of schizophrenia was associated with a decreased likelihood of a recorded diagnosis of a retinal disease across all age cohorts (Fig. 1a). Specifically, the adjusted RR was 0.90 for the age 50–59 cohort (95% CI 0.83, 0.96), 0.87 for the age 60–69 cohort (95% CI 0.83, 0.92), 0.91 for the age 70–79 cohort (95% CI 0.85, 0.97), and 0.82 for the age 80–89 cohort (95% CI 0.74, 0.91). Unadjusted and adjusted RR for specific retinal conditions in individuals with a recorded diagnosis of schizophrenia can be found in Supplementary Table 3.

After PSM of the BD cohorts, there were 160,921 individuals in the age 50–59 cohort; 141,638 individuals in the age 60–69 cohort; 67,028 individuals in the age 70–79 cohort; and 21,853 individuals in the age 80–89 cohort. The unadjusted RR of having a recorded diagnosis of a retinal disease in individuals with recorded diagnosis of BD was 1.81 compared to individuals without a recorded diagnosis of BD (95% CI 1.79, 1.83). After age stratification and PSM, a recorded diagnosis of BD was associated with an increased likelihood of a recorded diagnosis of a retinal disease in the age 60–69, 70–79, and 80–89 cohorts (Fig. 1b). Specifically, the adjusted RR was 1.04 for the age 60–69 cohort (95% CI 1.01, 1.08), 1.15 for the age 70–79 cohort (95% CI 1.10, 1.20), and 1.15 for the age 80–89 cohort (95% CI 1.07, 1.23). Unadjusted and adjusted RR for specific retinal conditions in individuals with a recorded diagnosis of BD can be found in Supplementary Table 4.

Among individuals with a recorded diagnosis of MDD, there were 645,256 matched individuals in the age 50–59 cohort; 658,244 in the age 60–69 cohort; 444,866 in the age 70–79 cohort; and 214,014 in the age 80–89 cohort. The unadjusted RR of having a recorded diagnosis of a retinal disease in individuals with a recorded diagnosis of MDD was 4.22 compared to individuals without a recorded diagnosis of MDD (95% CI 4.20, 4.23). Even after adjusting for confounders, a recorded diagnosis of MDD was associated with an increased likelihood of a recorded diagnosis of a retinal disease across all age cohorts (Fig. 1c). Specifically, the adjusted RR was 1.84 for the age 50–59 cohort (95% CI 1.79, 1.89), 1.70 for the age 60–69 cohort (95% CI 1.67, 1.74), 1.67 for the age 70–79 cohort (95% CI 1.64, 1.70), and 1.62 for the age 80–89 cohort (95% CI 1.58, 1.66). The unadjusted and adjusted RR for specific retinal conditions in individuals with a recorded diagnosis of MDD can be found in Supplementary Table 5.

### Risk of visual impairment

After propensity score matching, there were 8880 individuals in the schizophrenia and retinal disease cohort; 22,678 individuals in the BD and retinal disease cohort; and 265,544 individuals in the MDD and retinal disease cohort. The unadjusted RR of visual impairment diagnosis in individuals with a recorded diagnosis of schizophrenia and a retinal disease was 1.90 compared to individuals with a recorded diagnosis of a retinal disease alone (95% CI 1.81, 1.99). After matching, the adjusted RR of visual impairment diagnosis in individuals with a recorded diagnosis of schizophrenia and a retinal disease was 1.35 (95% CI 1.23, 1.48) (Table 1). The unadjusted RR of visual impairment diagnosis in individuals with a recorded diagnosis of BD and a retinal disease was 1.60 (95% CI 1.55, 1.65) compared to individuals with a recorded diagnosis of only a retinal disease. The adjusted RR of visual impairment diagnosis in individuals with a recorded diagnosis of BD and a retinal disease was 1.33 (95% CI 1.25, 1.42) (Table 2). Lastly, the unadjusted RR of visual impairment diagnosis in individuals with a recorded diagnosis of MDD and a retinal disease was 1.98 compared to individuals with a recorded diagnosis of a retinal disease alone (95% CI 1.96, 2.00). After matching, the adjusted RR of visual impairment diagnosis in individuals with a recorded diagnosis of MDD and a retinal disease was 1.51 (95% CI 1.48, 1.54) (Table 3).

**Table 1:** Relative risk of visual impairment in individuals with schizophrenia and retinal disease compared to individuals with a retinal disease alone after propensity score matching.

Schizophrenia			
Retinal disease	# Matched	Unadjusted RR (95% CI)	Adjusted RR (95% CI)
Diabetic retinopathy	3829	1.55 (1.43, 1.67)	1.38 (1.19, 1.60)
Chorioretinal scars	521	1.80 (1.52, 2.13)	1.60 (1.15, 2.23)
Retinal detachment and breaks	1053	2.89 (2.60, 3.21)	1.37 (1.13, 1.65)
Retinal vascular occlusions	533	1.88 (1.60, 2.20)	1.12 (0.86, 1.46)
Neovascular AMD	226	1.63 (1.28, 2.07)	1.94 (1.11, 3.38)
Non-neovascular AMD	631	1.37 (1.16, 1.62)	1.54 (1.04, 2.27)
Macular cyst, hole, or pseudohole	335	1.72 (1.35, 2.19)	1.06 (0.68, 1.67)
Cystoid macular degeneration	349	1.74 (1.40, 2.16)	1.33 (0.93, 1.91)
Degenerative drusen of macula	567	1.55 (1.25, 1.92)	1.18 (0.76, 1.85)
Puckering of macula	799	1.97 (1.69, 2.31)	1.21 (0.90, 1.64)
Peripheral retinal degeneration	539	2.31 (1.93, 2.77)	1.32 (0.89, 1.94)
Retinal hemorrhage	373	1.71 (1.41, 2.08)	1.17 (0.82, 1.68)
Separation of retinal layers	122	2.14 (1.49, 3.07)	1.36 (0.65, 2.85)
Retinal edema	465	2.10 (1.76, 2.51)	1.25 (0.91, 1.72)
Total retinal issue	8880	1.90 (1.81, 1.99)	1.35 (1.23, 1.48)

*ICD-10* International Statistical Classification of Diseases and Related Health Problems, Tenth Revision, *RR* risk ratio, *CI* confidence interval, *AMD* age-related macular degeneration.

**Table 2:** Relative risk of visual impairment in individuals with bipolar disorder and retinal disease compared to individuals with a retinal disease alone after propensity score matching.

Bipolar disorder			
Retinal disease	# Matched	Unadjusted RR (95% CI)	Adjusted RR (95% CI)
Diabetic retinopathy	8557	1.39 (1.32, 1.47)	1.18 (1.07, 1.30)
Chorioretinal scars	1190	1.62 (1.44, 1.82)	1.40 (1.12, 1.76)
Retinal detachment and breaks	2830	2.17 (2.02, 2.32)	1.31 (1.14, 1.50)
Retinal vascular occlusions	1480	1.89 (1.71, 2.08)	1.34 (1.13, 1.60)
Neovascular AMD	613	1.52 (1.29, 1.78)	1.56 (1.15, 2.12)
Non-neovascular AMD	1885	1.22 (1.09, 1.37)	1.28 (1.02, 1.61)
Macular cyst, hole, or pseudohole	789	1.94 (1.66, 2.26)	0.99 (0.73, 1.34)
Cystoid macular degeneration	870	1.84 (1.61, 2.10)	1.22 (0.95, 1.56)
Degenerative drusen of macula	1792	1.38 (1.21, 1.58)	1.43 (1.07, 1.91)
Puckering of macula	2736	1.83 (1.68, 2.00)	1.35 (1.13, 1.61)
Peripheral retinal degeneration	1521	1.51 (1.34, 1.71)	1.31 (1.00, 1.73)
Retinal hemorrhage	1013	1.60 (1.42, 1.81)	1.17 (0.92, 1.49)
Separation of retinal layers	388	1.74 (1.40, 2.15)	1.05 (0.70, 1.58)
Retinal edema	1093	1.75 (1.56, 1.97)	1.26 (1.02, 1.56)
Total retinal issue	22678	1.60 (1.55, 1.65)	1.33 (1.25, 1.42)

*ICD-10* International Statistical Classification of Diseases and Related Health Problems, Tenth Revision, *RR* risk ratio, *CI* confidence interval, *AMD* age-related macular degeneration.

**Table 3:** Relative risk of visual impairment in individuals with major depressive disorder and retinal disease compared to individuals with a retinal disease alone after propensity score matching.

Major depressive disorder			
Retinal disease	# Matched	Unadjusted RR (95% CI)	Adjusted RR (95% CI)
Diabetic retinopathy	93,902	2.08 (2.04, 2.12)	1.55 (1.51, 1.60)
Chorioretinal scars	12,681	2.05 (1.91, 2.14)	1.39 (1.29, 1.49)
Retinal detachment and breaks	32,586	2.30 (2.24, 2.36)	1.46 (1.40, 1.53)
Retinal vascular occlusions	20,261	2.16 (2.09, 2.22)	1.33 (1.27, 1.40)
Neovascular AMD	12,212	1.79 (1.72, 1.86)	1.50 (1.39, 1.62)
Non-neovascular AMD	32,059	1.96 (1.90, 2.01)	1.47 (1.38, 1.55)
Macular cyst, hole, or pseudohole	10,773	1.97 (1.87, 2.08)	1.48 (1.35, 1.62)
Cystoid macular degeneration	12,042	2.26 (2.17, 2.36)	1.38 (1.29, 1.49)
Degenerative drusen of macula	26,014	1.70 (1.64, 1.77)	1.36 (1.26, 1.46)
Puckering of macula	40,818	2.19 (2.13, 2.26)	1.35 (1.29, 1.42)
Peripheral retinal degeneration	16,665	2.11 (2.02, 2.21)	1.46 (1.34, 1.60)
Retinal hemorrhage	13,397	2.14 (2.05, 2.23)	1.41 (1.32, 1.52)
Separation of retinal layers	4781	2.27 (2.11, 2.43)	1.32 (1.16, 1.50)
Retinal edema	14,803	2.31 (2.22, 2.40)	1.41 (1.33, 1.51)
Total retinal issue	265,544	1.98 (1.96, 2.00)	1.51 (1.48, 1.54)

*ICD-10* International Statistical Classification of Diseases and Related Health Problems, Tenth Revision, *RR* risk ratio, *CI* confidence interval, *AMD* age-related macular degeneration.

The retinal disease with the highest adjusted RR of a recorded diagnosis of visual impairment in individuals with a recorded diagnosis of schizophrenia and BD was neovascular AMD at 1.94 (95% CI 1.11, 3.38) and 1.56 (95% CI 1.15, 2.12), respectively (Tables 1–2). For individuals with a recorded diagnosis of MDD, the retinal disease with the highest adjusted RR of a recorded diagnosis of visual impairment was DR at 1.55 (95% CI 1.51, 1.60) (Table 3). Additional RRs of visual impairment diagnosis for individuals with a recorded diagnosis of both a psychiatric and retinal condition can be found in Tables 1–3.

It is important to note that the associations noted in the study might relate to a variety of reasons which include a genuine association between a retinal and psychiatric disease through anatomical, genetic, and environmental risk factors and a genuine association between treatment of a psychiatric disorder and occurrence of retinal disease through psychotropic medications. Just as importantly, artefactual associations such as diagnostic variations across psychiatric disorders must be considered.

## Discussion

This study utilized a large national database and examined individuals over 50 years old who were diagnosed with a psychiatric disorder and retinal disease. In summary, we found that across each age cohort, recorded diagnoses of schizophrenia was associated with a decreased likelihood of recorded diagnoses of a retinal disease, while recorded diagnoses of BD and MDD was associated with an increased likelihood of recorded diagnoses of a retinal disease. However, concurrent diagnoses of a psychiatric disorder and retinal disease was associated with an increased likelihood of recorded diagnoses of visual impairment compared to individuals with a retinal disease alone.

Findings from existing studies suggest that psychiatric disorders may increase the risk of developing retinal disease by inducing anatomical and functional changes in the eye [21]. The literature acknowledges that individuals with schizophrenia, BD, and MDD exhibit a progressive loss of brain gray matter, and a growing body of emerging studies have begun to demonstrate that these populations also show retinal thinning on optical coherence tomography (OCT) scans and abnormal b-waves on electroretinogram (ERG) [13, 14, 22–31]. Anatomically, because the retina is a direct extension of the brain tissue, it is plausible that the pathological mechanisms that mediate the loss of the brain tissue in psychiatric disorders could potentially exacerbate retinal degeneration and subsequent vision loss [32]. This degeneration may be caused by the imbalance of neurotransmitters—such as dopamine, serotonin, and glutamate—that occurs in psychiatric disorders, as neurotransmitter balance is crucial for not only retinal cell functions but also for maintaining the homeostasis of the ocular environment and thus cell survival [33–39]. Genetic factors may also play a role in inducing the anatomical changes of the retina in individuals with psychiatric disorders, potentially increasing the likelihood of developing a retinal disease. Rabe et al. determined that genetic susceptibility to schizophrenia revealed significant associations with retinal thinning while in individuals with BD, Ayik et al. demonstrated through their familial study that GCL and IPL thickness may be a suitable endophenotype candidate for the mood disorder [40, 41].

It is possible that this finding reflects an artefactual association, as individuals with schizophrenia may be less likely to be evaluated and diagnosed with a retinal condition due to poorer healthcare follow-up and greater executive function impairments in this population compared to those with BD and MDD [42–46]. In addition, individuals with schizophrenia are more likely to encounter obstacles in navigating healthcare services due to increased discrimination and financial limitations, which are not as prominent in individuals with BD and MDD [47, 48]. Furthermore, individuals with BD and MDD on average access care more readily in part because these conditions are more often recognized in primary care settings. Because schizophrenia management is reliant on more specialized services, which are less accessible in many regions, this can lead to fewer referrals to ophthalmology by primary care compared to individuals with a mood disorder [48, 49]. As the average life expectancy in individuals with schizophrenia is also shorter than in individuals with BD and MDD, the decreased likelihood of recorded retinal disease diagnoses in individuals with schizophrenia may also be explained by a Neyman bias [50, 51].

On the other hand, the associations observed in this study may also have underlying physiological mechanisms. For instance, dopamine, which is the main neurotransmitter that is increased in schizophrenia, may serve as a protective factor against retinal diseases in these individuals, as dopamine has been reported to have protective anti-oxidant properties and contribute to the regulation of the retinal circadian rhythm [52–58]. This hypothesis is supported by the decreased dopamine levels seen in individuals with MDD and this study’s finding that a recorded diagnosis of MDD is associated with an increased likelihood of being diagnosed with a retinal disease [59, 60]. In regard to the increased likelihood of recorded retinal disease diagnoses in individuals with BD, potential explanations include structural abnormalities. Individuals with BD have been noted to have a thinner peripapillary RNFL and combined ganglion cell layer and inner plexiform layer (GCIPL), features that have been implicated in retinal detachment as well as other neurodegenerative retinal diseases [15, 61–64].

Another potential explanation for the increased likelihood of retinal disease diagnoses in individuals with psychiatric disorders includes the use of psychotropic medications. Typical antipsychotics including thioridazine and chlorpromazine have been shown to cause pigmentary retinopathy which involves oxidative stress and damage to the retinal pigmental epithelium [65]. Atypical antipsychotics including olanzapine and aripiprazole, however, have been associated with retinal vein occlusion and retinal degeneration respectively [66]. Lastly, medications used for MDD including selective serotonin reuptake inhibitors (SSRIs) and serotonin-norepinephrine reuptake inhibitors (SNRIs) have been linked to ischemic disorders of the retina and optic nerve head especially in individuals with a history of atherosclerosis [67]. While individuals with schizophrenia are typically on psychotropic medications, artefactual associations such as poorer access to care in this population likely outweigh medication effects and could explain the decreased likelihood of receiving a retinal disease diagnosis.

However, across each psychiatric disorder, individuals with a retinal disease had an increased risk of visual impairment compared to individuals with a retinal disease alone. Factors including neurotransmitter dysregulation and structural abnormalities, that may increase the likelihood of receiving a retinal disease diagnosis could also play a role in exacerbating disease progression and promoting visual impairment [15, 37–39, 61–64]. Artefactual reasons that need to be considered include ocular treatment regimen non-adherence and lack of health-care follow-up, resulting in poor disease management and worse visual outcomes [68–72].

Individuals with psychiatric disorders are often considered a vulnerable population due to systemic, social, and biologic factors that contribute to worse health outcomes compared to the general population [4, 73, 74]. Psychiatric disorders are also associated with comorbidities such as obesity, diabetes, and metabolic syndrome that are known to increase risk of retinal disease such as diabetic retinopathy [75–77]. Our findings suggest the need for closer monitoring of retinal disease and visual impairment in individuals with psychiatric disorders.

Although this network provides us with a substantial sample size to explore the relationship between psychiatric disorders and retinal disease, it is not without limitations. This study is limited by its retrospective nature and potential variations in coding practices for retinal diseases among clinicians and institutions. As the ICD-10 codes for diagnoses of low vision or blindness in this study may not be consistently used in practice, this study’s results are likely an underestimation of the true extent of visual impairments. OCT and other visual outcome data such as visual acuity were also unavailable in the network database. Moreover, unaccounted differences in variables—such as medication usage, duration of psychiatric disorders, lifestyle factors like smoking, socioeconomic status, and healthcare access—between different cohorts may have introduced confounding effects that we were not able to control for. In addition, it is important to note that visual impairment has been suggested to exacerbate the progression of certain psychiatric disorders [78–82]. Future studies should take these confounders into consideration and are needed to better ascertain the extent to which the association between psychiatric and retinal disorders is attributed to genetic and physiologic causes as opposed to structural factors.

In conclusion, this exploratory retrospective cohort study highlights that while individuals with recorded diagnoses of schizophrenia were less likely to have recorded diagnoses of a retinal disease, individuals with recorded diagnoses of BD and MDD were more likely. However, across all psychiatric disorders, individuals with a concurrent retinal disease were more likely to have visual impairment compared to individuals with a retinal disease alone. Ultimately, these findings suggest closer ophthalmology monitoring may be warranted for patients with psychiatric conditions.

Supplementary material is available at Eye’s website.

## Summary

### What was known before

- While patients with psychiatric disorders have been reported to have structural variations within the retina, the extent to which this puts patients at risk for retinal disease and vision loss has not been thoroughly investigated

### What this study adds

- Patients with schizophrenia are associated with a decreased risk of having a retinal disease while BD and MDD patients are associated with an increased risk.
- However, across all these psychiatric disorders, patients with a retinal disease may be at risk for increased visual loss compared to patients with a retinal disease alone.

## Supplementary information

Supplemental Table 1

Supplemental Table 2

Supplemental Table 3

Supplemental Table 4

Supplemental Table 5

## Data Availability

All data generated or analysed during this study are included in this published article.
