# Supplementary material for: Risk of retinal disease and visual impairment in individuals with psychiatric disorders
Source: Eye (Lond). 2025 May 20;39(11):2269–76. doi: 10.1038/s41433-025-03851-w (PMC12274455; doi:10.1038/s41433-025-03851-w)
Supplement: Supplementary file 1 — Supplemental Table 1 [file 41433_2025_3851_MOESM1_ESM.docx]

**Supplemental Table 1:** International Statistical Classification of Diseases and Related Health Problems, Tenth Revision (ICD-10) Codes

AMD = age-related macular degeneration
