# Supplementary material for: Risk of retinal disease and visual impairment in individuals with psychiatric disorders
Source: Eye (Lond). 2025 May 20;39(11):2269–76. doi: 10.1038/s41433-025-03851-w (PMC12274455; doi:10.1038/s41433-025-03851-w)
Supplement: Supplementary file 2 — Supplemental Table 2 [file 41433_2025_3851_MOESM2_ESM.docx]

**Supplemental Table 2:** Demographic Table Comparing Individuals with and without Psychiatric Disorders from TriNetX.

BD = bipolar disorder; MDD = major depressive disorder; sd = standard deviation

^a^Some data not reported.

^b^Race and ethnicity were determined according to designations within the electronic health record.
