# Supplementary material for: Risk of retinal disease and visual impairment in individuals with psychiatric disorders
Source: Eye (Lond). 2025 May 20;39(11):2269–76. doi: 10.1038/s41433-025-03851-w (PMC12274455; doi:10.1038/s41433-025-03851-w)
Supplement: Supplementary file 4 — Supplemental Table 4 [file 41433_2025_3851_MOESM4_ESM.docx]

**Supplemental Table 4:** Relative Risk of Having a Retinal Disease in Individuals with Bipolar Disorder Compared to Individuals without Bipolar Disorder After Age Stratification and Propensity Score Matching

CI = confidence interval; AMD = age-related macular degeneration
