# Supplementary material for: Risk of retinal disease and visual impairment in individuals with psychiatric disorders
Source: Eye (Lond). 2025 May 20;39(11):2269–76. doi: 10.1038/s41433-025-03851-w (PMC12274455; doi:10.1038/s41433-025-03851-w)
Supplement: Supplementary file 5 — Supplemental Table 5 [file 41433_2025_3851_MOESM5_ESM.docx]

**Supplemental Table 5:** Relative Risk of Having a Retinal Disease in Individuals with Major Depressive Disorder Compared to Individuals without Major Depressive Disorder After Age Stratification and Propensity Score Matching

CI = confidence interval; AMD = age-related macular degeneration
